# Massive intestinal ischemia, a rare complication of oral contraceptive-induced mesenteric venous thrombosis: a case report and review of literature

**DOI:** 10.1186/1757-1626-2-7416

**Published:** 2009-05-18

**Authors:** Eleni I Efremidou, George Kouklakis, Olga Tsirogianni, Nikolaos Courcoutsakis, Konstantinos J Manolas, Nikolaos Liratzopoulos

**Affiliations:** 1 First Surgical Department, University Hospital of Alexandroupolis, Democritus University of ThraceGreece; 2 Endoscopy Unit, University Hospital of Alexandroupolis, Democritus University of ThraceGreece; 3 Radiology Department, University Hospital of Alexandroupolis, Democritus University of ThraceGreece

## Abstract

Intestinal necrosis and perforation is a clinical and pathological presentation of the infrequently seen mesenteric venous thrombosis in women using oral contraceptives.

We report a case of a previously healthy 31-year-old female patient, who presented with a 3-day history of abdominal pain.

Although chest and abdomen radiographs showed small bowel obstruction, conservative treatment failed and the patient developed peritonism. Contrast-enhanced Tomography of the abdomen revealed free air associated with dilated and thickened small bowel. A laparotomy was performed and segmental resection of both small and large bowel was required. The pathological examination showed intestinal ischemia and mesenteric venous thrombosis. There were no further predisposing factors and mesenteric venous thrombosis was ascribed to oral contraceptives.

## Introduction

Oral contraceptives are widely used for birth control, treatment of dysmenorrheal, metromenorrhagia, endometriosis and suppression of postpartum lactation. Despite their use worldwide, contraceptives still carry an increased risk of several serious conditions such as venous thrombosis, pulmonary embolism, myocardial infraction, thrombotic stroke and hepatic adenomas and cholestatic jaundice [1, 2].

## Case presentation

A white 31-year old woman with Greek nationality was referred to Surgical Emergency Department with a 3-day history of abdominal pain. The pain was located in the left lower quadrant and characterized as intense. She denied fever, nausea, vomiting, diarrhea or constipation, hematochezia or melena. She had a surgical history of knee arthroplasty and benign breast tumor excision.

She did not smoke or drink alcohol. She was placed on oral contraceptive (0.02 mg ethinyl estradiol plus 0.075 mg gestodene) 3 months prior to presentation. Her medical and family history was unremarkable.

On examination she had a temperature of 38,2°C and tenderness in the periumbicial region and left lower quadrant. Rectal examination was normal.

The white blood cell count was 14.3x10^3^/ml and C-reaction protein was 7.8. Otherwise she had normal values of hematocrit, platelet count, amylase and liver function tests. Her prothrombin time was 13.3 seconds (normal range 10,1 to 12,9), partial thrombosis time was 35,4 seconds (normal range 24,00 to 35,00), and her international normalized ratio was 1.21(normal range 0,68 to 1,17).

A urine pregnancy test was negative. Chest radiograph was normal and abdomen series revealed scattered air fluid levels with no free air. Abdominal ultrasound showed small pelvic fluid collection. Conservative treatment failed and within 6 hours symptoms worsened and the patient developed peritonism. Contrast-enhanced Tomography (CT) of the abdomen and pelvis revealed dilation and thickening of the small intestine, small pelvic fluid collection and free air without an obvious obstruction.

Laparotomy was performed the same day which revealed peritonitis with ischemic necrosis of distal small bowel, gangrene of ileum with perforation plus ischemic necrosis of descending and sigmoid colon. After resection of 104 of distal jejunum and proximal ileum an end-to-end anastomosis was performed. Additionally a segment of 50 cm of descending and sigmoid colon was excised, the rectum was oversewn and a descending colostomy was fashioned.

Histopathologic examination of intestinal specimens showed a full thickness ischemic necrosis of both segments of small and large intestine and ileal perforation. The necrosis was accompanied by ulcerations of mucosa extending into the muscularis propria and mesenteric fat and thick cellular infiltration of polymorphonuclear. Vessels' hyperemia and diffused hemorrhagic perfusions co-existed. Respectively to the mesenterium, thrombosis of blood vessels was reported.

Postoperatively the patient received intravenous antibiotics, total parenteral nutrition and tinzaparin sodium 4500 IU/day subcutaneous. She was admitted in ICU just after laparotomy and was transferred in the clinic on postoperative day 10.Hematologic investigations included prothrombin time (11,1 s, normal range 10,1 to 12,9), partial thrombosis time (31,5 s, normal range 24,00 to 35,00), international normalized ratio (1,01 s, normal range 0,68 to 1,17) and antithrombin III (1,13 U/ml, normal range 0,86 to 1,20) while protein C was within normal range. There was no evidence of a congenitally inherent hypercoagulable state.

The patient was discharged home on postoperative day 18 on a 6-month course of tinzaparin sodium. She was seen in clinic 2, 4 weeks and 6 months after discharge and was doing well. Consequently, she is scheduled for convergence of colostomy.

## Discussion

Oral contraceptives (OCP) became available for use in the early 1960s. However, not long after their introduction complications of OCP became known. The most serious adverse effects are related to distribution along major vascular trunks (celiac, superior and inferior mesenteric) to the side of thrombus in either the arterial or the venous system.

The first report of a thromboembolic phenomenon (pulmonary embolus) associated to the use of OCP was published by Jordan in 1961, while the first report of MVT associated with OCP-use was published by Reed and Coon in 1963 [1].

Thrombosis caused by OCP can be venous or arterial in the pulmonary, systemic or splanchnic circulation. Estrogen component of OCP is associated with both arterial and venous occlusion, while progestin is related only with arterial occlusion [3].

OCP-relative MVT accounts for 4-5% of total MVT. Whereas smoking, hypertension, age, connective tissue disease and the dose of estrogenic fraction are correlated with thrompogenicity, the duration of OCP is not a predisposing factor [3, 4].

Several pathophysiologic mechanisms have been proposed to explain the relationship between OCP and “hypercoagulable state”:

(i) estrogens increase the procoagulant factors like prothrombin, VII, X, XII and XIII (ii) estrogens have an effect on anticoagulant pathway by causing resistance to activated protein C (iii) estrogens increase fibrinolysis (iv) progestin exacerbate the effects of estrogens on procoagulant, anticoagulant and fibrinolytic pathways [3, 4].

This multifactorial set of conditions that favors vascular narrowing or occlusion, seems to be the explanation for the association between intestinal ischemia and oral contraceptives.

Complications of OCP ranging from enterocolitis to intestinal perforation and peritonitis are mentioned in the international literature.

Since 1986 Schneiderman et al. reported in a review study a total of 15 patients suffering from enterocolitis/colitis as a complication of OCP use, who were treated conservatively. In the same study was reported that 26 women appeared with OCP-associated bowel infraction, from whom 25 underwent laparotomy. From the latter 22 patients underwent segmental intestinal resection due to necrosis or perforation, while free peritoneal fluid or fecal contamination was infrequently reported [1].

The review of more recently published data revealed further five cases of OCP-associated intestinal ischemia [3, 5-8] and one case of intravaginal contraceptive-associated intestinal ischemia [4]; in two of them conservative treatment with heparin was successful [4, 8], while in four patients a laparotomy was required [3, 5-7] and segmental bowel resection was performed in three of them [3, 6, 7].

Nevertheless, up to date, only four cases of OCP-associated MVT causing intestinal necrosis and perforation are reported in the literature [2, 7, 9].

The first report concerns a young woman on OCP for a 6-month period who underwent laparotomy and segmental resection of infracted ileum due to MVT; semipurulent fluid and fibrinous peritonitis were observed in the abdomen [9].

Koh et al. reported two cases of female smokers, who were on OCP for 5 and 8 years respectively and developed MTV, intestinal perforation and acute abdomen. One of the above-mentioned patients underwent segmental resection of terminal ileum and sigmoid colon, while in the second patient an extensive ileal resection was performed [2].

The latter case report concerns an adolescent girl who was on a small dose of OCP for hirsutism and developed small bowel ischemia with three perforations and localized abscesses. Resection of the involved intestine was required [7].

As far as our patient is concerned, it appears to be the 4^th^ reported case of intestinal perforation due to MVT associated with OCP use.

The patient received OCP for a 3-month period only, without having any other thrombosis predisposition and without having been a smoker. Despite all these, she rapidly developed an extensive necrosis of small intestine and also of sigmoid colon, intestinal perforation and fecal peritonitis. A wide resection, involving 104 cm of ileum and jejunum and 50 cm of descending and sigmoid colon was required, being the second reported segmental excision of both small and large bowel, associated with OCP-caused MVT [2]. Postoperatively the patient was on LMWH (Low Molecular Weight Heparin) for 6 months, although the benefit of anticoagulation has not been proved.

## Conclusion

Intestinal ischemia and necrosis caused by mesenteric venous thrombosis is one of the rarer cardiovascular complications of oral contraceptives, which carry a high mortality rate. However, the number of reported cases is small and there have been no controlled studies. Therefore, this association is noteworthy for all clinicians who should consider intestinal ischemia in women on pill developing sudden severe abdominal pain without any other obvious cause of pathology.

## List of abbreviations

CRP: C-reaction protein
CT: Contrast-enhanced Tomography
INR: international normalized ratio
ICU: Intensive care unit
LMWH: Low Molecular Weight Heparin
MVT: Mesenteric Venous Thrombosis
OCP: Oral contraceptives
PT: Prothrombin time
PTT: Partial thrombosis time
